# Comparison of cognitive profiles in spinocerebellar ataxia subtypes: a case series

**DOI:** 10.1186/s40673-019-0107-4

**Published:** 2019-09-18

**Authors:** Corey Bolton, Maureen Lacy

**Affiliations:** Department of Psychiatry and Behavioral Neurosciences, University of Chicago Medical Center, 5841 S. Maryland Ave, Chicago, IL 60637 USA

**Keywords:** Cerebellum, Spinocerebellar ataxias, Neuropsychology, Cognition, Depression

## Abstract

**Background:**

The spinocerebellar ataxias (SCA) are a heterogeneous group of progressive neurodegenerative disorders that are associated with diffuse cerebellar atrophy. While the physical symptoms of this condition have long been studied, more attention has been given to cognitive changes in recent years. We describe a case series of four adults with various genetically-confirmed subtypes of SCA.

**Case presentation:**

Patients with SCA types 2, 3, and 6 presented with impaired cognitive profiles consistent with the existing literature while the reported patient with SCA-14 showed notable impairment inconsistent with the only published case controlled study.

**Conclusions:**

Comparisons were made between the four patients with a common pattern of slowed processing speed, poor memory retrieval, and reduced mental flexibility. Confrontation naming and consolidation-based memory were intact across all patients. These findings are discussed in light of the relevant literature on cerebellar cognitive affective syndrome.

## Background

The spinocerebellar ataxias (SCA) are a group of progressive neurodegenerative disorders marked by ataxia and a variety of other neurologic features with atrophy predominantly in the cerebellum. There is significant heterogeneity between the unique subtypes with regard to the pattern of neurodegeneration and subsequent clinical features. There has been much recent interest in the nonmotor functions of the cerebellum, stemming primarily from the proposed cerebellar cognitive affective syndrome (CCAS [1];). SCAs, with their notable cerebellar involvement, typically are associated with cognitive deficits; however, there is little understanding as to typical neurocognitive profiles associated with each subtype. Disruption of cerebrocerebellar circuitry has been posited as a mediator of the relationship between SCA and cognitive impairment, though given the heterogeneity of pathology in SCA, some differences in neurocognitive functioning have been noted across the various subtypes [2]. Cognitive impairment in SCA seems to correlate with the severity of physical symptoms, suggesting that the extent of cerebellar dysfunction is directly related to neurocognitive functioning [3]. The following cases highlight key differences in neurocognitive presentation between four different genetically-confirmed subtypes of SCA. Table 1 lists neuropsychological measures used and summarizes neurocognitive profiles for each patient. Standardized scores were obtained from commonly used normative data with corrections for age and, as available, other demographic variables such as education, gender, and ethnicity.

**Table 1:** Summary of neuropsychological assessment data by subtype of SCA

			Case 1	Case 2	Case 3	Case 4
SCA Subtype			2	3	6	14
Age (years)			66	53	70	46
Disease Duration (years)			40	12	25	3
SARA Score			9	21	18.5	11
Global	MMSE [4]		–	–	–	–
Verbal Memory	Verbal List (RBANS or CVLT-II) [5, 6]	Immediate Recall	+++	+	++	–
		Delayed Recall	++	–	++	–
		Recognition	+++	–	–	+
	Verbal Story (RBANS or WMS-IV) [5, 7]	Immediate Recall	++	–	–	–
		Delayed Recall	++	–	–	–
		Recognition	+++	–	–	–
Visual Memory	Simple (BVMT-R) [8]	Immediate Recall	–	++	+	++
		Delayed Recall	–	++	–	++
		Recognition	–	–	+	+
	Complex (RCFT) [9]	Immediate Recall	/	–	/	++
		Delayed Recall	/	–	/	++
		Recognition	/	–	/	++
Visuospatial	Line Orientation (RBANS or JLO) [5, 10]		–	–	–	–
	Clock Copy [11]		–	+	–	–
Language	Boston Naming Test [12]		–	–	–	–
	Verbal Fluency [13]	Phonemic	–	+++	–	++
		Semantic	+	++	++	–
Executive Functions Attention and Processing Speed	WCST [14]	Errors	–	–	–	+
		Perseverative Responses	+	+	–	–
		Categories Completed	+	–	+	+
	Trail Making Test Part B [15]		++	++	+	+
	Trail Making Test Part A [15]		++	+	+	+
	Symbol-Digit Coding (RBANS or SDMT) [5, 16]		+	++	+	+
Depression	Screening Measure (BDI-II or GDS) [17, 18]		+	++	–	–

*+ = Mildly Impaired, ++ = Moderately Impaired, +++ = Severely Impaired, − = Within Normal Limits, / = Not assessed. All comparisons are with normative data. SCA2 = spinocerebellar ataxia type 2, SCA3 = spinocerebellar ataxia type 3, SCA6 = spinocerebellar ataxia type 6, SCA14 = spinocerebellar ataxia type 14. MMSE = Mini Mental State Examination; RBANS = Repeatable Battery for the Assessment of Neuropsychological Status; CVLT-II = California Verbal Learning Test – Second Edition; WMS-IV = Wechsler Memory Scale – Fourth Edition; BVMT-R = Brief Visuospatial Memory Test – Revised; RCFT = Rey Complex Figure Test; JLO = Judgement of Line Orientation; WCST = Wisconsin Card Sorting Test; SDMT = Symbol Digit Modalities Test; BDI-II = Beck Depression Inventory – Second Edition; GDS = Geriatric Depression Scale*

## Case presentation

### Case 1 – SCA2

A 66-year-old African-American female with 14 years of education presented with slowly progressive imbalance, dysarthria, and dysmetria over the last 40 years. More recently she began to note mild memory problems. Neurological exam was notable for wide-based, ataxic gait, mild dysarthria, slowed eye movements, and abnormal finger-nose-finger and alternating movements. The Scale for the Assessment and Rating of Ataxia (SARA) total score was 9. Neuropsychological assessment revealed grossly intact cognition on screening (Mini-Mental State Examination; MMSE = 27) with more in-depth assessment revealing notable difficulties in verbal learning, processing speed, mental flexibility, speeded semantic fluency and problem solving deficits. Responses to a self-report measure also revealed mild acute clinical depressive symptoms. Visual memory, visuospatial and constructional abilities, confrontational naming, and phonemic fluency were as expected for age and education.

### Case 2 – SCA3

A 53-year-old Caucasian male with 12 years of education presented with a 12-year history of progressive imbalance, slurred speech, and hand incoordination with no reported cognitive difficulties. Additionally, he reported urinary incontinence, diplopia, and dysarthria. He began using a walker 7 years ago and now requires a wheelchair. Neurological exam was notable for square wave jerks, moderate dysarthria, wide-based ataxic gait, abnormal finger-nose-finger with intention tremor, abnormal alternating movements, and absent reflexes in biceps, brachioradialis, knee jerks, and ankle jerks. Total SARA score was 21. Neuropsychological assessment revealed grossly intact cognition upon screening (MMSE = 28) with further evaluation revealing deficits in memory retrieval for visual and verbal material, along with processing speed, mental flexibility, speeded semantic fluency, and speeded phonemic fluency deficits. On a self- report questionnaire, he endorsed symptoms indicative of a clinical depression in the moderate range of severity. Visuospatial perception, memory consolidation and object naming were within normal limits.

### Case 3 – SCA6

A 70-year-old Caucasian female with 16 years of education presented with imbalance, dysarthria, and dyscoordination that has progressed over the last 25 years. Neurological exam revealed abnormal eye movements, mild to moderate dysarthria, intention tremor, abnormal alternating movements and finger-nose-finger, and wide-based ataxic gait. The SARA total score was 18.5. Neuropsychological assessment revealed grossly intact cognition on screening (MMSE = 27) with more in-depth assessment revealing difficulties in memory retrieval, processing speed, semantic fluency, and mental flexibility. Visuospatial perception, visuoconstructional abilities, object naming, and phonemic fluency were within normal limits, as were scores on a depression screener.

### Case 4 – SCA14

A 46-year-old Caucasian male with 12 years of education presented with a 3 year history of progressive dysarthria, unsteady gait, vision problems, and cognitive deficits. He also reported a history of seizures and slow speech dating back to childhood. Neurological exam revealed abnormal eye movements, mild dysarthria, abnormal finger-nose-finger and heel-knee-shin, mild intention tremor, and ataxic gait. The SARA total score was 11. Neuropsychological assessment revealed notable deficits in visual learning, processing speed, and speeded phonemic fluency. Performances in tests of verbal memory, semantic fluency, object naming, and visuoconstructional abilities were as expected for age and education. On a self-report questionnaire, the patient endorsed only minimal depressive symptoms.

## Discussion

We describe a case series of adults with various genetically-confirmed subtypes of SCA. All cases presented with characteristic ataxic gait, ocular motor abnormalities, dysarthria, and uncoordinated movement upon neurological examination. Ataxia severity was variable with SARA scores ranging from 9 to 21. These symptoms are consistent with diffuse cerebellar atrophy. While it has long been recognized that the cerebellum serves a critical role in movement, nonmotor symptoms of cerebellar dysfunction have been increasingly realized in recent years.

Past studies have shown that SCA subtypes 2, 3, and 6 are often associated with a frontal-subcortical pattern of neurocognitive impairment. Primarily, deficits have been described in attentional abilities and executive functions [2, 3, 19–26]. The patients in this report with the more common SCA subtypes of 2, 3, and 6 presented with very similar neurocognitive profiles as previously described. SCA14, the rarest of the four presented subtypes, has received very little attention with regard to the associated cognitive profile. The one case controlled study that has been reported found very little neurocognitive impairment in these patients [27]. Our patient, however, displayed notable deficits in visual memory, processing speed, and speeded phonemic fluency. It is notable, however, that this patient had limited premorbid intellect and a history of childhood seizures, thus increasing vulnerability and making generalizable conclusions about the effects of SCA on his cognition difficult to obtain.

Comparison of the four patients in this case series highlights the distinct cognitive profiles associated with various subtypes of SCA, as supported by larger past studies [2, 3, 19–27]. While some heterogeneity exists, there were some notable commonalities as well. Across all patients, processing speed was notably slowed. This is consistent with recent findings of slowed processing speed in patients with reductions in gray matter in the posterior lobule of the cerebellum [28]. Additionally, all patients displayed learning or memory retrieval deficits, with no one exhibiting pure memory consolidation deficits. Verbal fluency was reduced for all patients, though the modality (phonemic vs. semantic) was inconsistent. Finally, all patients displayed mental flexibility impairment consistent with frontal circuity disruption.

The deficits observed in this small sample of SCA patients were consistent with the CCAS with deficits in executive functioning, memory retrieval and aspects of language abilities [29]. One notable difference amongst this sample is that no patients in the present study were found to have even a mild anomia. In the defining study of CCAS, 13 of 20 patients experienced difficulties with confrontation naming, though it was noted that naming was spared in those with smaller lesions [1]. It was suggested that naming deficits in CCAS may represent impaired lexical access, as patients appear to improve with phonemic cueing [30]. It is likely, therefore, that naming deficits may only be present in late stage SCA, once cerebellar-prefrontal networks have been sufficiently degraded to inhibit lexical access.

## Conclusions

SCA is a progressive neurodegenerative disease that selectively targets the cerebellum. Given the recent exploration of the cerebellum’s role in cognition, the neurocognitive profile of this disease is of interest. This study provided support for the growing literature of the neurocognitive profiles associated with SCA2, SCA3, and SCA6, while also providing the first report of a pattern of deficits in SCA14. Amongst all four subtypes, slowed processing speed was found. This likely reflects the important role of the cerebellum in efficient information processing. Further, it was noted that no patients were anomic, a finding contrary to that expected from the CCAS literature and likely indicative of less advanced disease.

## Data Availability

All data generated or analyzed during this study are included in this published article in Table 1. Raw data scores are available from the corresponding author upon request.

## Abbreviations

CCAS: cerebellar cognitive affective syndrome
MMSE: Mini-Mental State Examination
SARA: Scale for the Assessment and Rating of Ataxia
SCA: spinocerebellar ataxia
